# Rates of respiratory syncytial virus (RSV)-associated hospitalization among adults with congestive heart failure—United States, 2015–2017

**DOI:** 10.1371/journal.pone.0264890

**Published:** 2022-03-09

**Authors:** Stephanie A. Kujawski, Michael Whitaker, Matthew D. Ritchey, Arthur L. Reingold, Shua J. Chai, Evan J. Anderson, Kyle P. Openo, Maya Monroe, Patricia Ryan, Erica Bye, Kathryn Como-Sabetti, Grant R. Barney, Alison Muse, Nancy M. Bennett, Christina B. Felsen, Ann Thomas, Courtney Crawford, H. Keipp Talbot, William Schaffner, Susan I. Gerber, Gayle E. Langley, Lindsay Kim

**Affiliations:** 1 Epidemic Intelligence Service, Centers for Disease Control and Prevention (CDC), Atlanta, GA, United States of America; 2 Division of Viral Diseases, National Center for Immunization and Respiratory Diseases, Centers for Disease Control and Prevention (CDC), Atlanta, GA, United States of America; 3 Eagle Global Scientific, Atlanta, GA, United States of America; 4 Division for Heart Disease and Stroke Prevention, National Center for Chronic Disease Prevention and Health Promotion, Centers for Disease Control and Prevention (CDC), Atlanta, GA, United States of America; 5 US Public Health Service, Rockville, MD, United States of America; 6 Division of Epidemiology, School of Public Health, University of California, Berkeley, CA, United States of America; 7 California Emerging Infections Program, Oakland, CA, United States of America; 8 Career Epidemiology Field Officer, Center for Preparedness and Response, Centers for Disease Control and Prevention, Atlanta, GA, United States of America; 9 Departments of Medicine and Pediatrics, Emory University School of Medicine, Atlanta, GA, United States of America; 10 Georgia Emerging Infections Program, Atlanta, GA, United States of America; 11 Atlanta Veterans Affairs Medical Center, Atlanta, GA, United States of America; 12 Foundation for Atlanta Veterans Education and Research, Decatur, GA, United States of America; 13 Maryland Department of Health, Baltimore, MD, United States of America; 14 Minnesota Department of Health, St. Paul, MN, United States of America; 15 New York State Department of Health, Albany, NY, United States of America; 16 University of Rochester School of Medicine and Dentistry, Rochester, NY, United States of America; 17 Public Health Division, Oregon Health Authority, Portland, OR, United States of America; 18 Vanderbilt University Medical Center, Nashville, TN, United States of America; Carol Davila University of Medicine and Pharmacy: Universitatea de Medicina si Farmacie Carol Davila, ROMANIA

## Abstract

**Background:**

Respiratory syncytial virus (RSV) can cause severe disease in adults with cardiopulmonary conditions, such as congestive heart failure (CHF). We quantified the rate of RSV-associated hospitalization in adults by CHF status using population-based surveillance in the United States.

**Methods:**

Population-based surveillance for RSV (RSV-NET) was performed in 35 counties in seven sites during two respiratory seasons (2015–2017) from October 1–April 30. Adults (≥18 years) admitted to a hospital within the surveillance catchment area with laboratory-confirmed RSV identified by clinician-directed testing were included. Presence of underlying CHF was determined by medical chart abstraction. We calculated overall and age-stratified (<65 years and ≥65 years) RSV-associated hospitalization rates by CHF status. Estimates were adjusted for age and the under-detection of RSV. We also report rate differences (RD) and rate ratios (RR) by comparing the rates for those with and without CHF.

**Results:**

2042 hospitalized RSV cases with CHF status recorded were identified. Most (60.2%, n = 1230) were ≥65 years, and 28.3% (n = 577) had CHF. The adjusted RSV hospitalization rate was 26.7 (95% CI: 22.2, 31.8) per 10,000 population in adults with CHF versus 3.3 (95% CI: 3.3, 3.3) per 10,000 in adults without CHF (RR: 8.1, 95% CI: 6.8, 9.7; RD: 23.4, 95% CI: 18.9, 28.5). Adults with CHF had higher rates of RSV-associated hospitalization in both age groups (<65 years and ≥65 years). Adults ≥65 years with CHF had the highest rate (40.5 per 10,000 population, 95% CI: 35.1, 46.6).

**Conclusions:**

Adults with CHF had 8 times the rate of RSV-associated hospitalization compared with adults without CHF. Identifying high-risk populations for RSV infection can inform future RSV vaccination policies and recommendations.

## Introduction

While respiratory syncytial virus (RSV) is a common cause of lower respiratory tract infection in infants and children [1], it can also cause severe disease in older adults[2, 3] and adults with cardiopulmonary conditions, such as congestive heart failure (CHF) [4, 5]. RSV infection has been associated with hospitalizations and deaths in adults with cardiopulmonary conditions [3, 5–8]. However, most studies to date examining the association between RSV and cardiopulmonary conditions have been among older adult populations [3, 4, 6], and less is known about risk across the adult age spectrum.

Currently, there are several clinical trials for RSV vaccines and monoclonal antibodies in progress [9]. Understanding disease burden and identifying potential high-risk populations for RSV infection can inform RSV vaccination policies and recommendations [10]. The objective of this analysis was to quantify the rate of RSV infection in hospitalized adults by CHF status and age (<65 years and ≥65 years) using a population-based surveillance platform in the United States.

## Methods

### Surveillance population and data collection

The Centers for Disease Control and Prevention (CDC) performs population-based surveillance in the United States for RSV through the Emerging Infections Program’s RSV-NET, a network of state and local health departments, acute care hospitals, and academic institutions. Surveillance was performed over two respiratory seasons (2015–2017), from October 1–April 30 each year, at seven sites (California, Georgia, Maryland, Minnesota, New York, Oregon [2016–2017 only], and Tennessee). These sites encompassed 35 counties, with an annual catchment population of approximately 13.7 million adults (4.2% of the U.S. population). Adults (≥18 years) admitted to a catchment area hospital with laboratory-confirmed RSV infection within 14 days of admission or any time during hospitalization were included in the study. RSV was diagnosed through clinician-directed testing. Demographic and clinical information, including presence of underlying CHF, was abstracted from medical records by trained surveillance officers.

### Statistical analysis

RSV-NET data were entered into a Microsoft Access database or REDCap at each site and cleaned and analyzed in SAS version 9.4 and Stata version 14 SE. We calculated descriptive statistics (frequency distribution and median, where applicable) for all RSV cases and stratified by CHF status. Demographic and clinical characteristics by CHF status were compared using chi-square tests or Wilcoxon rank sum tests, where appropriate.

We calculated incidence rates (per 10,000 population) of RSV by CHF status using hospitalized RSV cases as the numerator and RSV-NET county-level population estimates as the denominator; both numerator and denominator were stratified by CHF status. Projected county-level population estimates for 2016 and 2017 were derived from the 2010 U.S. Census [11]. To estimate the population with diagnosed CHF in the catchment area, we multiplied the population by the estimated prevalence of CHF by county. For adults ≥65 years, county-level CHF prevalence was obtained from 2016 and 2017 Centers for Medicare and Medicaid Services (CMS) data [12]. CMS calculates CHF prevalence based on inpatient and outpatient ICD diagnosis codes from fee-for-service beneficiaries with claims data available [13]. To obtain county-level CHF prevalence estimates for adults <65 years, we took the ratio of self-reported prevalence of CHF of adults <65 years to adults ≥65 years from the 2015–2016 National Health and Nutrition Examination Survey (NHANES), a nationally representative survey, and then applied this ratio to the CMS prevalence [14]. At the time of the analysis, the 2015–2016 NHANES was the most recent NHANES dataset available. Clopper-Pearson 95% confidence intervals for CHF prevalence by age group were calculated from NHANES and applied to the county-level estimates. We also report rate differences (RD) and rate ratios (RR) by comparing the rates for those with and without CHF.

RSV-associated hospitalization rates were adjusted for the under-detection of RSV to account for the sensitivities of the laboratory tests used to diagnose RSV and for clinician testing practices. Methods for adjusting rates for under-detection are described elsewhere [15]. Briefly, to determine the under-detection of RSV, on a monthly basis, the surveillance sites identify all hospitalized patients who had an acute respiratory infection (based on ICD-10 pneumonia and influenza codes; 115,623 hospitalized patients over the surveillance period) and randomly selected 10 patients from each of five age groups (18–49, 50–64, 65–74, 75–84, ≥85). For each patient selected, the sites determine whether the patient was tested for RSV, and if so, the type of test used (polymerase chain reaction assay [PCR] or rapid antigen). From these data, we estimated the probability of being detected as: Probability (testing for RSV)*Probability (sensitivity of the RSV tests) = Probability (detection). Test sensitivities were calculated as a weighted average of the types of tests performed multiplied by their individual sensitivities (PCR: 95%, rapid antigen: 29%) [16, 17]. We multiplied the age-specific probability of testing proportions by that age groups’ distribution in the RSV-NET population. The sum of those two products was the weighted (or age-adjusted) probability of testing. Crude hospitalization rates were then adjusted by the inverse of the probability of detection (P(detection)), referred to as the under-detection multiplier. RSV-associated hospitalization rates were then calculated per season and averaged. For all ages (≥18 years), rates were adjusted for age by standardizing to the overall catchment population distribution of adults <65 years and ≥65 years. Rates were also stratified by age groups of interest (<65 vs. ≥65 years).

#### Ethics statement

This activity was reviewed by CDC and was conducted consistent with applicable federal law and CDC policy. (See e.g., 45 C.F.R. part 46.102(l)(2), 21 C.F.R. part 56; 42 U.S.C. §241(d); 5 U.S.C. §552a; 44 U.S.C. §3501 et seq.) Participating sites obtained approval for the RSV-NET surveillance protocol from their respective state and local institutional review boards, as needed. CDC waived informed consent for the surveillance protocol. All data had unique study identifiers that were assigned prior to data transfer and analysis from sites to CDC. Patients’ medical records were accessed by sites between October 2016–December 2017. Retrospective data were accessed for the 2015–2016 respiratory season during this aforementioned time period.

## Results

During the 2015–2017 surveillance seasons, 2204 hospitalized RSV cases were identified, of which 2042 (92.6%) had a CHF status recorded and were therefore used as the analytic sample. Most (60.2%, n = 1230) were ≥65 years and were female (59.2%, n = 1208) (Table 1). About 41% (n = 833) had more than one form of insurance, among whom 63.6% (n = 530) had both Medicare and private insurance. The median length of hospital stay was 4 (IQR: 3–7) days, and 20.3% (n = 414) were admitted to the intensive care unit (ICU) during their stay. Overall, 4.9% (n = 101) died during hospitalization.

**Table 1:** Adults testing positive for RSV by CHF status, RSV-NET, 2015–2017 (N = 2042).

	TOTAL	CHF	No CHF	p-value^a^
	(N = 2042)	(N = 577)	(N = 1465)	
	No. (%)	No. (%)	No. (%)	
**Demographics**		** **		** **
Age (years), median (IQR)	69 (57–82)	76 (64–86)	67 (54–79)	<0.001
<65 years	812 (39.8)	153 (26.5)	659 (45.0)	<0.001
≥65 years	1230 (60.2)	424 (73.5)	806 (55.0)	
Sex				
Male	834 (40.8)	245 (42.5)	589 (40.2)	0.35
Female	1,208 (59.2)	332 (57.5)	876 (59.8)	
COPD	656 (32.1)	230 (39.9)	426 (29.1)	<0.001
Asthma	524 (25.7)	142 (24.6)	382 (26.1)	0.50
Immunocompromising condition^b^	481 (23.6)	99 (17.2)	382 (26.1)	<0.001
Number of underlying conditions, median (range)^c^	2 (0–6)	3 (1–6)	2 (0–6)	<0.001
Race				
White	1,298 (63.6)	370 (64.1)	928 (63.3)	0.12
Black or African American	434 (21.3)	135 (23.4)	299 (20.4)	
Asian/Pacific Islander	153 (7.5)	37 (6.4)	116 (7.9)	
American Indian or Alaska Native	7 (0.3)	1 (0.2)	6 (0.4)	
Multiracial	10 (0.5)	0 (0)	10 (0.7)	
Not specified	140 (6.9)	34 (5.9)	106 (7.2)	
Body mass index				
Underweight (<18.5)	87 (4.3)	20 (3.5)	67 (4.6)	0.009
Normal (18.5–24.9)	492 (24.1)	118 (20.5)	374 (25.5)	
Overweight (25–29.9)	567 (27.8)	161 (27.9)	406 (27.7)	
Obese (30–39.9)	542 (26.5)	164 (28.4)	378 (25.8)	
Morbidly obese (≥40)	204 (10.0)	76 (13.2)	128 (8.7)	
*Missing*	150 (7.4)	38 (6.6)	112 (7.7)	
Insurance				
Private only	297 (14.5)	44 (7.6)	253 (17.3)	<0.001
Medicare only	433 (21.2)	129 (22.4)	304 (20.8)	
Medicaid only	154 (7.5)	32 (5.6)	122 (8.3)	
Other only	19 (0.9)	6 (1.0)	13 (0.9)	
>1 insurance	833 (40.8)	299 (51.8)	534 (36.4)	
No insurance	26 (1.3)	5 (0.9)	21 (1.4)	
Unknown	280 (13.7)	62 (10.7)	218 (14.9)	
Smoking status				
Current	368 (18.0)	80 (13.9)	288 (19.7)	0.001
Former	688 (33.7)	225 (39.0)	463 (31.6)	
No/unknown	986 (48.3)	272 (47.1)	714 (48.7)	
**Clinical course**				
Viral co-detection	130 (6.8)	40 (7.3)	90 (6.5)	0.52
Bacterial co-detection	145 (7.1)	47 (8.1)	98 (6.7)	0.25
Length of hospital stay, median (IQR)	4 (3–7)	5 (3–9)	4 (2–7)	<0.001
ICU admission	414 (20.3)	141 (24.5)	273 (18.6)	0.003
Mechanical ventilation	171 (41.3)	59 (41.8)	112 (41.0)	0.90
Length of ICU stay, median (IQR)	3 (1–7)	3 (2–9)	3 (1–6)	0.18
Died	101 (4.9)	45 (7.8)	56 (3.8)	<0.001

Abbreviations: CHF = congestive heart failure; ICU = intensive care unit; IQR = interquartile range; RSV respiratory syncytial virus.

^a^ chi-square test or Wilcoxon rank sum test, where appropriate.

^b^ Immunocompromising conditions included AIDS or CD4 count <200 cells/mm^3^, being in treatment for cancer or cancer diagnosed in the last 12 months, complement deficiency, HIV infection, immunoglobulin deficiency, immunosuppressive therapy, bone marrow transplant, organ transplant, steroid therapy, or other immunosuppressive condition.

^c^ Underlying conditions included chronic lung disease, chronic metabolic disease, blood disorders, cardiovascular disease, neurologic disease, renal disease, and liver disease.

Over a quarter of hospitalized RSV cases (28.3%, n = 577) had CHF, the majority of whom were ≥65 years (73.5% vs. <65 years: 26.5%) (Table 1). Hospitalized RSV cases with CHF were older, with a median age of 76 years vs. 67 years (p<0.001) and were more likely to also have chronic obstructive pulmonary disease (COPD) (39.9% vs. 29.1%, p<0.001) and be obese or morbidly obese (41.6% vs. 34.5%) compared with hospitalized RSV cases without CHF. Similar proportions had asthma (CHF: 24.6%, no CHF: 26.1%, p = 0.50), while a lower proportion of adults with CHF had an immunocompromising condition (CHF: 17.2%, no CHF: 26.1%, p<0.001). A higher proportion of cases with CHF were admitted to the ICU (24.5% vs. 18.6%, p = 0.003) and a higher proportion died during their hospitalization (7.8% vs. 3.8%, p<0.001) compared with those without CHF. Similar trends in background characteristics and clinical course by CHF status were observed when stratified by ages <65 and ≥65 (S1 Table).

**Fig 1:**
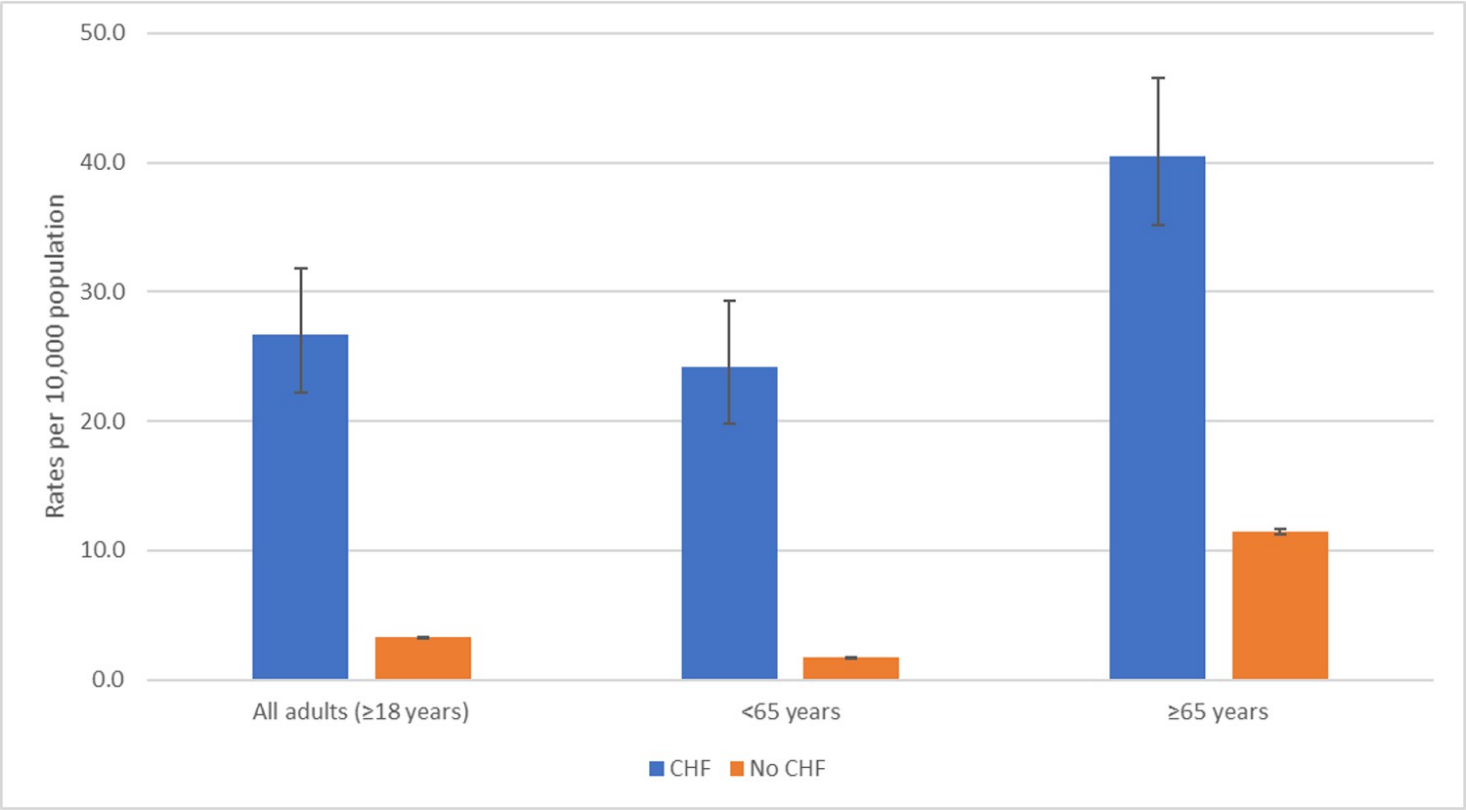
Adjusted rates^a^ (per 10,000 population) of RSV-associated hospitalization by congestive heart failure (CHF) status, RSV-NET, 2015–2017 (N = 2042). ^a^Adjusted for age and the under-detection of RSV.

When examining surveillance data to determine under-detection rates, the proportion of patients tested for RSV was similar in the <65 years age group (19.8%) and the ≥65 years age group (19.2%). Accounting for testing and sensitivity of the test, the under-detection multipliers were 5.3 and 5.5 for adults <65 years and adults ≥65 years, respectively (S2 Table). Adjusting for age and under-detection, the rate of RSV hospitalization was 26.7 (95% CI: 22.2, 31.8) per 10,000 population in adults with diagnosed CHF compared with 3.3 (95% CI: 3.3, 3.3) per 10,000 in adults without diagnosed CHF (Fig 1, Table 2). When stratified by age (<65 years vs. ≥65 years), in both age groups, those with CHF had higher rates of RSV hospitalization than those without CHF. The highest rates were in adults ≥65 years with CHF (40.5 per 10,000 population, 95% CI: 35.1, 46.6). Rates did not vary greatly by year (S3 Table).

**Table 2:** Rates (per 10,000 population) of RSV-associated hospitalization by congestive heart failure (CHF) status, RSV-NET, 2015–2017 (N = 2042).

	Crude				Adjusted for under-detection^a^ and age							
	CHF rate	95% CI	no CHF rate	95% CI	CHF rate	95% CI	no CHF rate	95% CI	Rate difference	95% CI	Rate ratio	95% CI
**All adults (≥ 18 years)**	6.3	5.4, 7.4	0.6	0.6, 0.6	26.7	22.2, 31.8	3.3	3.3, 3.3	23.4	18.9, 28.5	8.1	6.8, 9.7
**<65 years**	4.5	3.7, 5.5	0.3	0.3, 0.3	24.1	19.8, 29.3	1.7	1.7, 1.7	22.5	18.1, 27.6	14.3	11.8, 17.3
**≥65 years**	7.4	6.4, 8.5	2.1	2.0, 2.1	40.5	35.1, 46.6	11.4	11.2, 11.7	29.1	23.9, 34.9	3.5	3.1, 4.0

^a^ Adjusted for the under-detection of RSV (based on clinician testing practices and sensitivities of the laboratory tests).

Overall, for all ages, there were 23.4 (95% CI:18.9, 28.5) additional cases of hospitalization with RSV per 10,000 population among those with CHF compared to those without CHF (Table 2, Fig 1). Adults with CHF had 8.1 (95% CI: 6.8, 9.7) times the rate of RSV hospitalizations compared with adults without CHF. When stratified by age group, in adults ≥65 years with CHF, the rate of RSV hospitalization was 3.5 (95% CI: 3.1, 4.0) times that of adults without CHF (RD: 29.1, 95% CI: 23.9, 34.9). In adults <65 years with CHF, the RSV rate was 14.3 (95% CI: 11.8, 17.3) times the rate in adults without CHF (RD: 22.5, 95% CI: 18.1, 27.6).

## Discussion

Using population-based surveillance, we found that adults with CHF had 8 times the rate of RSV hospitalizations compared with adults without CHF. This corresponds to an excess of 23 additional cases of RSV per 10,000 population. While rates of RSV-associated hospitalizations were highest among adults ≥65 years with CHF (40.5 per 10,000 population), rates were also high in adults <65 years with CHF (24.1 per 10,000 population). These results highlight the increased hospitalization burden of RSV in adults with CHF across age groups, which is important for informing decisions about RSV prevention and treatment interventions among adults with CHF.

To our knowledge, this is one of the first studies to calculate rates of RSV hospitalization in all adults by CHF status. A recent study compared RSV-associated acute respiratory illness hospitalization rates among adults with and without CHF in New Zealand, similarly finding increased rates among those with CHF in all age groups (18–49 years: 11.22 per 10,000 adults; 50–64 years: 7.93 per 10,000 adults) and the highest rate in adults with ≥65 years with CHF (13.74 per 10,000 adults) [18]. Our rates in the ≥65 years age group were substantially higher, which may be due to important study differences. The New Zealand study did not correct for under-detection, and the majority of enrolled patients were required to meet the World Health Organization severe acute respiratory illness case definition, defined as cough and measured or reported fever. Requiring fever in the case definition might lead to underestimation of the burden of hospitalized RSV cases as fever is infrequently reported among older adults [19]. Other studies, while not directly comparable, have described RSV infections in hospitalized adults [20, 21], older adults [4, 21, 22], and among adults with cardiopulmonary disease more generally [4, 5]. Similar to other studies, we found RSV rates in those with CHF increased with age [20–22] and were higher compared to healthy elderly adults [5] or to those without these cardiopulmonary conditions [4, 18].

In addition to having higher risk for RSV-associated hospitalizations, a higher proportion of adults with CHF had >1 insurance type. This may indicate that those with CHF may need insurance coverage due to their underlying condition. Further, adults with CHF were also more frequently admitted to the ICU and a higher proportion died. Similar proportions of ICU admission for RSV among older adults have been reported in other studies [3, 23]. Similarly, in one study among adults with RSV, adults ≥60 years of age with CHF had more moderate to severe disease and were more likely to have a serious outcome (defined by the study as hospitalization, emergency department visit, or pneumonia) than those without CHF [4]. Poor health outcomes, including mechanical ventilation, hospitalization, and death, have also been documented previously in adults with RSV and cardiopulmonary disease (e.g., CHF or COPD) or cardiac disease [2, 5]. Our finding that a high proportion of patients with CHF also had COPD is consistent with other studies describing pulmonary and cardiac disease comorbidities with RSV infection [3, 6, 24, 25]. Indeed, having both CHF and COPD might increase the risk for medically attended RSV infection [26]. Future analyses should consider assessing the separate and combined RSV burden with different cardiopulmonary conditions, as well as the impact of severity of cardiopulmonary conditions on clinical outcomes.

This analysis is subject to a few limitations. First, RSV cases were identified through clinician-directed testing. While we adjusted the rates for the potential under-detection of RSV due to testing practices and test sensitivity, this adjustment did not account for testing practices by CHF status. In another adult surveillance system, hospitalized patients with CHF were less likely to be tested for RSV than patients without CHF [27]. As such, the RSV rates for patients with CHF presented in this analysis may represent a lower bound of the true population rate. Second, diagnosed CHF prevalence was based on available Medicare claims data from CMS, which excluded enrollees with partial coverage or with Medicare Advantage (i.e. Medicare Part C: plans offered by Medicare-approved private companies). For adults <65 years, county-level CHF prevalence was not directly available and was estimated based on NHANES and CMS data. NHANES collects self-reported CHF status, which may be underestimated. Therefore, these data sources may not reflect the true county-level prevalence of CHF. Third, it is possible that RSV cases were not mutually exclusive; patients with CHF are often re-hospitalized. Fourth, as noted, a substantial proportion of the RSV cases had both CHF and COPD. Therefore, we were not able to determine the relative contribution of CHF and COPD to increased rates of RSV among those with both conditions. Lastly, because the surveillance system only collects data on laboratory-confirmed RSV hospitalizations, we were unable to create a non-RSV comparison group.

In conclusion, we found high rates of RSV hospitalization among adults of all ages with CHF. RSV should be considered as a cause of acute respiratory illness and as potentially contributing to CHF exacerbations in adults during the respiratory season. More research is needed on the role of RSV in acute exacerbations of CHF and the impact on long-term health outcomes. Understanding the importance of RSV in patients with CHF can also inform future vaccine and treatment recommendations.

## Supporting information

S1 Table Adults testing positive for RSV by CHF status and by age group, RSV-NET, 2015–2017 (N = 2042). (PDF)

S2 Table Estimated multipliers for under-detection of RSV in RSV-NET, 2015–2017. (PDF)

S3 Table Rates (per 10,000 population) of RSV-associated hospitalization by congestive heart failure (CHF) status and by surveillance year, RSV-NET, 2015–2017 (N = 2042). (PDF)
