# Supplementary material for: Rates of respiratory syncytial virus (RSV)-associated hospitalization among adults with congestive heart failure—United States, 2015–2017
Source: PLoS One. 2022 Mar 9;17(3):e0264890. doi: 10.1371/journal.pone.0264890 (PMC8906631; doi:10.1371/journal.pone.0264890)
Supplement: S1 Table — (PDF) [file pone.0264890.s001.pdf]

S1 Table. Adults testing positive for RSV by CHF status and by age group, RSV-NET, 2015–2017 (N = 2042)

	<65 years (n=812)				p-value[a]	≥65 years (n=1230)				p-value[a]
	CHF (n=153)		No CHF (n=659)			CHF (n=424)		No CHF (n=806)		
	No.	%	No.	%		No.	%	No.	%	
**Demographics**										
Sex										
Male	67	43.8	265	40.2	0.42	178	42.0	324	40.2	0.55
Female	86	56.2	394	59.8		246	58.0	482	59.8	
COPD	58	37.9	171	26.0	0.003	172	40.6	255	31.6	0.002
Asthma	52	34.0	193	29.3	0.25	90	21.2	189	23.4	0.38
Immunocompromising condition[b]	36	23.5	237	36.0	0.003	63	14.9	145	18.0	0.16
Number of underlying conditions, median (range)[c]	3	(2–3)	1	(1–2)	<0.001	3	(2–4)	2	(1–3)	<0.001
Race										
White	68	44.4	341	51.8	0.22	302	71.2	587	72.8	0.01
Black or African American	66	43.1	218	33.1		69	16.3	81	10.0	
Asian/Pacific Islander	6	3.9	31	4.7		31	7.3	85	10.5	
American Indian or Alaska Native	1	0.7	4	0.6		0	0.0	2	0.2	
Multiracial	0	0.0	8	1.2		0	0.0	2	0.2	
Not specified	12	7.8	57	8.7		22	5.2	49	6.1	
Body mass index										
Underweight (<18.5)	6	3.9	30	4.6	<0.001	14	3.3	37	4.6	0.22
Normal (18.5–24.9)	23	15.0	166	25.2		95	22.4	208	25.8	
Overweight (25–29.9)	31	20.3	161	24.4		130	30.7	245	30.4	
Obese (30–39.9)	47	30.7	177	26.9		117	25.6	201	24.9	
Morbidly obese (≥40)	39	25.5	81	12.3		37	8.7	47	5.8	
Missing	7	4.6	44	6.7		31	7.3	68	8.4	
Insurance										
Private only	26	17.0	211	32.0	<0.001	18	4.3	42	5.2	0.12
Medicare only	16	10.5	58	8.8		113	26.7	246	30.5	
Medicaid only	26	17.0	111	16.8		6	1.4	11	1.4	
Other only	1	0.7	6	0.9		5	1.2	7	0.9	
>1 insurance	60	39.2	148	22.5		239	56.4	386	47.9	
No insurance	3	2.0	18	2.7		2	0.5	3	0.4	

	<65 years (n=812)				p-value	≥65 years (n=1230)				p-value
	CHF (n=153)		No CHF (n=659)			CHF (n=424)		No CHF (n=806)		
	No.	%	No.	%		No.	%	No.	%	
Unknown	21	13.7	107	16.2		41	9.7	111	13.8	
Smoking status										
Current	44	28.8	219	33.2		36	8.5	69	8.6	
Former	45	29.4	150	22.8	0.20	180	42.5	313	38.8	0.45
No/unknown	65	41.8	290	44.0		208	49.1	424	52.6	
**Clinical course**										
Viral co-detection	10	7.1	48	7.9	0.77	30	7.4	42	5.5	0.19
Bacterial co-detection	13	8.5	54	8.2	0.90	34	8.0	44	5.5	0.08
Length of hospital stay, median (IQR)	6	(3–10)	4	(2–7)	<0.001	5	(3–8)	4	(2–6)	0.003
ICU admission	51	33.3	144	21.9	0.003	90	21.3	129	16.0	0.02
Mechanical ventilation	22	43.1	60	41.7	0.91	37	41.1	52	40.3	0.83
Length of ICU stay, median (IQR)	5	(2–14)	3	(1–7)	0.007	3	(1–6)	3	(1.5–6)	0.76
Died	5	3.2	18	2.7	0.72	40	9.4	48	4.7	0.001

Abbreviations: CHF = congestive heart failure; ICU = intensive care unit; IQR = interquartile range; RSV respiratory syncytial virus.

[a] chi-square test or Wilcoxon rank sum test, where appropriate

[b] Immunocompromising conditions included AIDS or CD4 count <200 cells/mm^3, being in treatment for cancer or cancer diagnosed in the last 12 months, complement deficiency, HIV infection, immunoglobulin deficiency, immunosuppressive therapy, bone marrow transplant, organ transplant, steroid therapy, or other immunosuppressive condition.

[c] Underlying conditions included chronic lung disease, chronic metabolic disease, blood disorders, cardiovascular disease, neurologic disease, renal disease, and liver disease.
