# Supplementary material for: Rates of respiratory syncytial virus (RSV)-associated hospitalization among adults with congestive heart failure—United States, 2015–2017
Source: PLoS One. 2022 Mar 9;17(3):e0264890. doi: 10.1371/journal.pone.0264890 (PMC8906631; doi:10.1371/journal.pone.0264890)
Supplement: S2 Table — (PDF) [file pone.0264890.s002.pdf]

S2 Table. Estimated multipliers for under-detection of RSV in RSV-NET, 2015–2017

	Multiplier	95% CI
Overall		
All ages	5.7	3.6-13.7
<65 years	5.3	3.4-12.0
≥65 years	5.5	3.5-13.2
**2015-16**		
All ages	6.8	4.0-23.4
<65 years	5.9	3.5-19.6
≥65 years	6.3	3.7-19.8
**2016-17**		
All ages	5.3	3.4-12.4
<65 years	4.9	3.2-10.5
≥65 years	4.5	3.0-9.1

Note: All ages multiplier is larger than age stratified multipliers due to beta binomial distribution adjustment.
