# Supplementary material for: Rates of respiratory syncytial virus (RSV)-associated hospitalization among adults with congestive heart failure—United States, 2015–2017
Source: PLoS One. 2022 Mar 9;17(3):e0264890. doi: 10.1371/journal.pone.0264890 (PMC8906631; doi:10.1371/journal.pone.0264890)
Supplement: S3 Table — (PDF) [file pone.0264890.s003.pdf]

S3 Table. Rates (per 10,000 population) of RSV-associated hospitalization by congestive heart failure (CHF) status and by surveillance year, RSV-NET, 2015–2017 (N = 2042)

	Crude				Adjusted for under-detection[a] and age				Adjusted for under-detection[a] and age			
	CHF (95% CI)		no CHF (95% CI)		CHF (95% CI)		no CHF (95% CI)		Rate difference	95% CI	Rate ratio	95% CI
**2015–2016**												
**All ages**	5.1	4.3, 6.0	0.5	0.5, 0.5	24.5	20.4, 29.2	3.2	3.2, 3.2	21.3	17.2, 26.0	7.7	6.4, 9.2
**<65 years**	3.7	3.1, 4.5	0.3	0.3, 0.3	22.1	18.1, 26.8	1.7	1.7, 1.7	20.3	16.4, 25.0	12.7	10.4, 15.3
**≥65 years**	5.9	5.1, 6.8	1.7	1.7, 1.7	36.8	31.9, 42.3	10.6	10.4, 10.8	26.2	21.6, 31.5	3.5	3.1, 3.9
**2016–2017**												
**All ages**	7.5	6.4, 8.8	0.7	0.7, 0.7	28.4	23.7, 33.9	3.4	3.4, 3.4	25.0	20.3, 30.6	8.4	7.0, 10.0
**<65 years**	5.3	4.4, 6.5	0.3	0.3, 0.3	25.9	21.2, 31.4	1.6	1.6, 1.6	24.3	19.6, 29.8	15.8	13.0, 19.1
**≥65 years**	8.8	7.7, 10.2	2.5	2.4, 2.5	39.5	34.2, 45.4	11.0	10.8, 11.2	28.5	23.5, 34.2	3.6	3.2, 4.0

[a] Adjusted for the under-detection of RSV (based on clinician testing practices and sensitivities of the laboratory tests).
